# The Attentional Suppressive Surround: Eccentricity, Location-Based and Feature-Based Effects and Interactions

**DOI:** 10.3389/fnins.2018.00710

**Published:** 2018-10-08

**Authors:** Sang-Ah Yoo, John K. Tsotsos, Mazyar Fallah

**Affiliations:** ^1^Department of Psychology, York University, Toronto, ON, Canada; ^2^Centre for Vision Research, York University, Toronto, ON, Canada; ^3^Active and Attentive Vision Laboratory, Department of Electrical Engineering and Computer Science, York University, Toronto, ON, Canada; ^4^Visual Perception and Attention Laboratory, School of Kinesiology and Health Science, York University, Toronto, ON, Canada

**Keywords:** attention, surround suppression, selective tuning, location-based attention, feature-based attention, vision, visual processing

## Abstract

The Selective Tuning model of visual attention (Tsotsos, 1990) has proposed that the focus of attention is surrounded by an inhibitory zone, eliciting a center-surround attentional distribution. This attentional suppressive surround inhibits irrelevant information which is located close to attended information in physical space (e.g., Cutzu and Tsotsos, 2003; Hopf et al., 2010) or in feature space (e.g., Tombu and Tsotsos, 2008; Störmer and Alvarez, 2014; Bartsch et al., 2017). In Experiment 1, we investigate the interaction between location-based and feature-based surround suppression and hypothesize that the attentional surround suppression would be maximized when spatially adjacent stimuli are also represented closely within a feature map. Our results demonstrate that perceptual discrimination is worst when two similar orientations are presented in proximity to each other, suggesting the interplay of the two surround suppression mechanisms. The Selective Tuning model also predicts that the size of the attentional suppressive surround is determined by the receptive field size of the neuron which optimally processes the attended information. The receptive field size of the processing neurons is tightly associated with stimulus size and eccentricity. Therefore, Experiment 2 tested the hypothesis that the size of the attentional suppressive surround would become larger as stimulus size and eccentricity increase, corresponding to an increase in the neuron's receptive field size. We show that stimulus eccentricity but not stimulus size modulates the size of the attentional suppressive surround. These results are consistent for both low- and high-level features (e.g., orientation and human faces). Overall, the present study supports the existence of the attentional suppressive surround and reveals new properties of this selection mechanism.

## Introduction

“The darkest place is under the candlestick.” means that people tend to overlook nearby things. Interestingly, this expression corresponds well to one aspect of the Selective Tuning model (ST) of visual attention—center-surround distribution of attention (Tsotsos, 1990; for a full specification see Tsotsos, 2011; for review, see Carrasco, 2011). Unlike other attention models, such as spotlight (Eriksen and Hoffman, 1973; Posner et al., 1980), zoom lens (Eriksen and Yeh, 1985; Eriksen and James, 1986), and gradient [LaBerge, 1983; LaBerge and Brown, 1986; Andersen and Kramer, 1993; Cheal et al., 1994; see also Bylinskii et al. (2015) and Rothenstein and Tsotsos (2014) for the extensive review of attention models], the center-surround distribution requires that the attentional focus is accompanied by a suppressive surround to contrast the attended and unattended information, thus, the attended information becomes more conspicuous. When visual information is spatially close to the attentional focus so it falls within the suppressive zone, its processing is inhibited, whereas visual information beyond the suppressive surround is not affected. Therefore, it elicits a Difference-of-Gaussians attentional profile (Tsotsos, 1990) where the surround of the attentional focus is attenuated but perceptual processing further away is unaffected.

**Figure 1:**
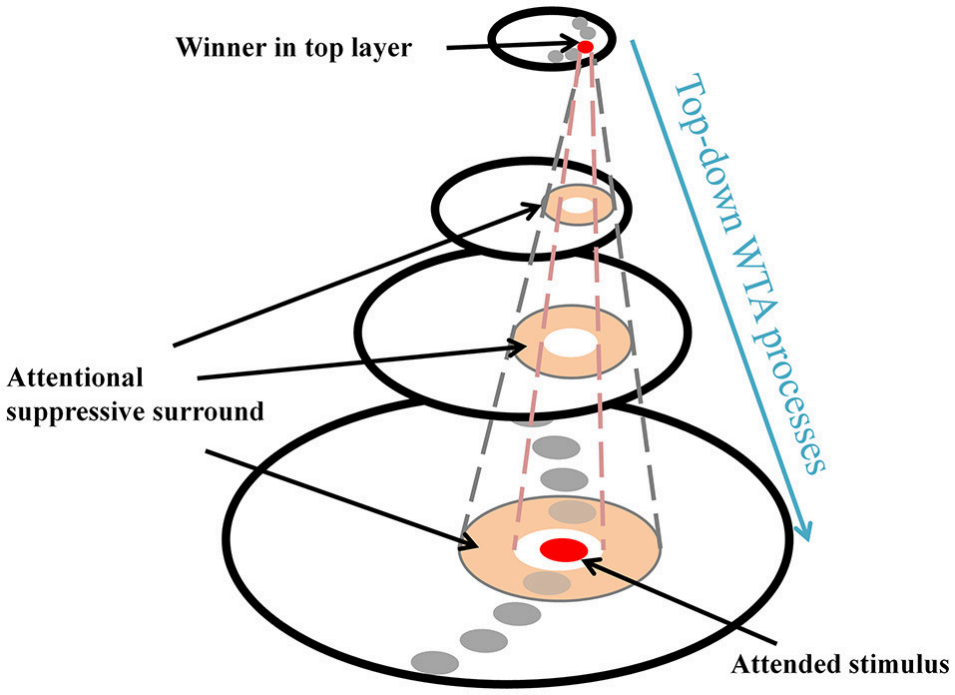
Schematic attentional selection as suggested by the Selective Tuning model. When a signal from an attended stimulus reaches the top layer, a top-down winner-take-all (WTA) mechanism localizes this stimulus. During this process, the mechanism inhibits irrelevant connections that surround the attended stimulus, thus, attentional surround suppression occurs (adapted from Hopf et al., 2006 with permission).

ST provides a theoretical explanation of the center-surround distribution which has been supported by substantial behavioral and physiological evidence (e.g., Cutzu and Tsotsos, 2003; Müller and Kleinschmidt, 2004; Müller et al., 2005; Hopf et al., 2006, 2010; Boehler et al., 2009, 2011; Bartsch et al., 2017). In everyday vision, a neuron's receptive field (RF) often “sees” more than a single object and this is more apparent in higher areas in the visual processing hierarchy due to increasing RF sizes. Thus, it is necessary to filter out irrelevant signals within the RF so that the visual system can isolate a stimulus-of-interest. ST posits that top-down winner-take-all (WTA) processes select the strongest inputs to neurons (winner) at each processing level, and then prune away losing input connections around the winner. Those pruned connections form a suppressive surround and it enhances processing of the attended stimulus, by inhibiting interference of nearby items. However, the other connections located far from the attended stimulus that do not interfere with the processing of the stimulus remain unaffected (Figure 1). In ST, this attentional modulation changes the center-surround structure of a neuron's classical RF where an attended stimulus lies (Tsotsos, 1990, 2011) and such major changes in RF structure also have been observed elsewhere (e.g., Womelsdorf et al., 2006). Note that the attentional surround suppression in ST, which is due to top-down influences rather than sensory, horizontal or lateral influences (see Hopf et al., 2010 and Cutzu and Tsotsos, 2003, for arguments), is different from surround suppression in which visual stimuli located outside of the classical RF modulate the neuron's response to stimuli within the RF (Ozeki et al., 2009; Haider et al., 2010; Adesnik et al., 2012; Self et al., 2014).

ST has made many predictions regarding the characteristics of the attentional surround suppression. Unlike other attentional models, no experimental data was incorporated into ST's design or theory. ST is a model derived from first principles based on computational complexity theory and more generally on other computational principles considered foundational to the computational understanding of solutions in machines or in nature (see Denning, 2007). Other models that are based more strongly on experimental data provide quantitative predictions but can do so largely because they perform some level of data-fitting so interpolation or extrapolation within data models is possible. ST has no such basis and hence, ST does not give quantitative predictions such as actual response times, neural response curves, or accuracies that can be directly compared to experimental results. Importantly, however, this also means it can give counter-intuitive predictions that are not of the same character as existing experiments (Braun et al., 2001). Several predictions of this kind have already been shown to lead to new knowledge about attentional processing (e.g., Carrasco, 2011) something which data-based models may not provide. It may be possible to combine ST's theoretical basis with some level of data fitting, but at this point, this is left for future work as it does not directly impact the conclusions of the current work.

**Figure 2:**
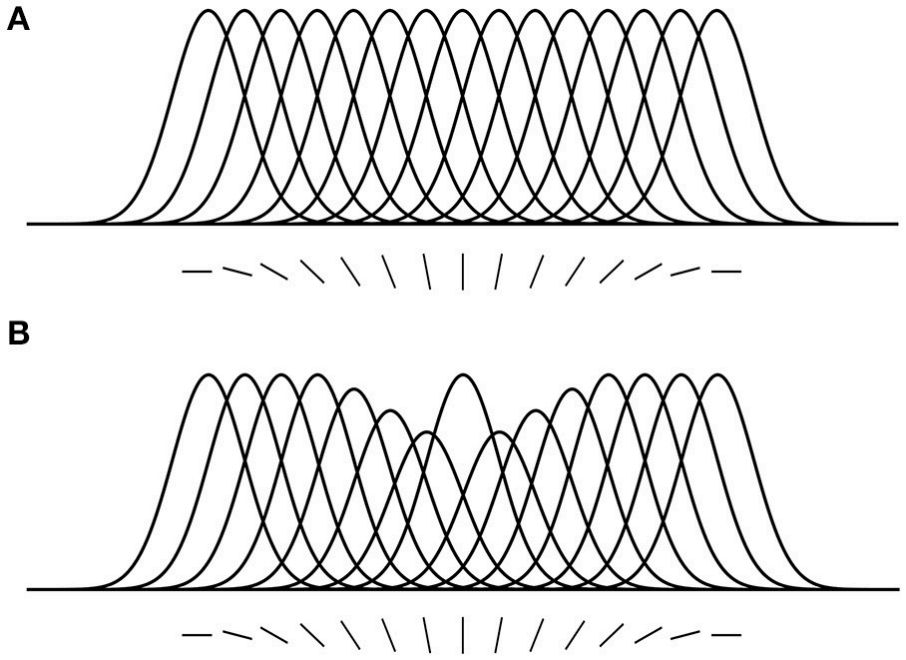
Feature-based attentional surround suppression in the orientation map. **(A)** Hypothetical tuning curves for different orientations. **(B)** Attention to the vertical orientation inhibits neighboring orientations and changes the overall tuning profile in the orientation map (adapted from Tsotsos, 2011 with permission).

Here, we introduce some predictions that are relevant to the hypotheses and findings of the current study. (1) Location-based attentional surround suppression occurs if and only if a task requires spatial localization of the attended stimulus (Boehler et al., 2009; Hopf et al., 2010). For example, a pop-out color detection task that can be performed without localization does not produce an attentional suppressive surround. In addition, the suppressive effect is manifested after 250 ms relative to stimulus onset, reflecting a top-down process through the visual hierarchy. (2) ST predicts that the size of the attentional suppressive surround would be determined by the neuron's RF size that best represents the attended stimulus (Tsotsos, 2011). Ever since the introduction of scale-space theory in the 1980's, it has been accepted that there is a best scale at which to represent visual objects. Given that neurons have scales of representation defined by their RF sizes, then there would be a best size for any given visual stimulus. A main element of ST is that attentive suppression helps reduce or eliminate the interference from unattended stimuli within a RF. Combining these, ST predicts that the size of the attentional suppressive surround should be related to the RF size of the neuron that best represents the attended stimulus and that the RF size should be closest to the size of the attended stimulus and large enough to include the whole stimulus. Motivated by this prediction, Hopf et al. (2010) hypothesized that the size of the attentional suppressive surround would differ depending on the processing level of the attended feature in visual hierarchy due to different neurons' RF sizes across visual areas. That is, a simple feature represented at a low level visual area would produce a narrower suppressive surround than a complex feature because of smaller RF size in the low level area. They, however, observed equally large suppressive surrounds for color- and luminance-targets, suggesting the earliest processing level of an attended feature may not influence the size of the attentional suppressive surround. A more appropriate test might have been to compare with more abstract targets (such as a face, or animal, that would require neural activations higher than V4) but this remains for future work. In addition, this experiment did not consider how RF sizes are represented throughout the visual hierarchy as well as across the visual field and as a result this experimental design did not test these factors. Since then, Kay et al. (2013) have elucidated this relationship and our current design appropriately adjusts the methodology. (3) ST proposed that an analogous suppressive mechanism operates in the feature domain as well (Tsotsos, 1990). For instance, orientation-selective neurons in area V1 are organized in a columnar structure (Hubel and Wiesel, 1968, 1974), and these neurons respond best to their preferred orientations but the responses gradually decrease as orientation becomes dissimilar from the preferred one, showing Gaussian-like tuning curves (Figure 2A). Once attention is directed to a certain orientation (e.g., vertical), “feature-based” attentional surround suppression attenuates the processing of neighboring orientations without affecting the processing of orientations that represented far from the attended orientation (Figure 2B). It eventually changes the overall population tuning curve in the orientation map into a center-surround structure, sharpening the tuning to the attended orientation by suppressing its surround. Evidence for feature-based surround suppression is observed in orientation (Tombu and Tsotsos, 2008), action (Loach et al., 2008), and color (Störmer and Alvarez, 2014; Bartsch et al., 2017) domains, as well as for features in visual working memory (Kiyonaga and Egner, 2016).

In the present study, we investigated the properties of attentional surround suppression that remain open. We set two research questions based on ST's original predictions and subsequent empirical findings. The first is whether the mechanisms of location-based and feature-based surround suppression interact with each other. Secondly, we explored what determines the size of the attentional suppressive surround, looking for empirical evidence for ST's prediction that the size of the attentional surround suppression is associated with the attended neuron's RF size (Tsotsos, 2011). To answer these questions, we conducted two experiments using a target discrimination task that has already been well established as an effective measurement of the attentional suppressive surround (Cutzu and Tsotsos, 2003). During the task, participants were asked to judge whether a cued target (reference target) and the second target (probe) were the same or different while the distance between them systematically varied. Here, an attentional cue guides attention to the location of the reference target and prioritizes its processing and the instruction to the participant to attend to that cue causes a suppressive surround around the reference target to develop. If a *nearby* probe falls within the suppressive surround, target discrimination performance is deteriorated due to the suppression. Indeed, Cutzu and Tsotsos showed that target discrimination accuracy was lowest when two targets were closest to each other but it improved when the inter-target distance increased, indicating location-based attentional surround suppression.

In Experiment 1, we hypothesized that the suppressive effects would be demonstrated more strongly when an attended stimulus and a nearby stimulus are located closely both in physical space and in a shared feature dimension if they indeed interact. Participants performed a similar task to the one in Cutzu and Tsotsos, but the stimuli were oriented bars instead of letters. As we hypothesized, we found that the center-surround attentional profile changes depending on the spatial and feature (i.e., orientation) distances between the target stimuli, indicating an interaction between location-based and feature-based surround suppression. Experiment 2 addressed whether stimulus size and eccentricity, which activate populations of neurons with correspondingly different RF sizes, led to differences in the size of the suppressive surround. Although the idea of the processing level of attended features has been considered previously without conclusion (Hopf et al., 2010), sufficient variation in RF sizes to enable the detection of differences was not included, something that could be done by varying stimulus eccentricity. Neurons' RF sizes vary depending on retinal eccentricity, increasing toward the periphery (Hubel and Wiesel, 1974; Wilson and Sherman, 1976; Smith et al., 2001). Kay et al. (2013) provide a thorough set of relationships among RF size, eccentricity and visual areas, demonstrating that the stimulus eccentricities used in Hopf et al. (2010) may have involved too small a variation in RF sizes to be an effective experimental probe. By varying stimulus eccentricity, we hypothesized that a stimulus presented in the periphery would produce a wider suppressive surround than a stimulus presented in the foveal region. In addition, when the size of a stimulus becomes larger, more neurons will be involved in representing each part of the stimulus. Hence, we predicted that increments of the stimulus size would enlarge the sum of those neurons' RF sizes and it would consequently make the size of the attentional suppressive surround larger. The current results suggest that stimulus eccentricity but not stimulus size changes the size of the attentional suppressive surround. This supports the portion of the original prediction that the surround size depends on the neuron RF that represents the target.

## Experiment 1

### Methods and materials

#### Participants

Thirty-eight York University students (ages 17–34, 29 female) participated in Experiment 1. All participants were unaware of the purpose of the experiment. They had normal or corrected-to-normal vision and normal color vision (tested with Ishihara Color Plates). Informed consent was obtained from all participants and participants recruited from Undergraduate Research Participants Pool (URPP) of York University received course credits for their study participation. The research was approved by York University's Human Participants Review Committee.

#### Apparatus and stimuli

Participants performed the experiments in a dark room. The position of each participant's head was stabilized by a head and chin rest (Headspot, UHCOtech) placed 57 cm from a CRT monitor (21″ View Sonic G225f, 1280x1024, 85 Hz). Each participant wore an infrared eye tracker (Eyelink II, SR Research, 500 Hz) monitoring the left eye position. The stimuli were created using MATLAB (The Mathworks Corp.) and the Psychophysics Toolbox (Brainard, 1997; Pelli, 1997). Experimental control was maintained by Presentation (Neurobehavioral Systems). Data analyses were conducted using MATLAB and SPSS (IBM).

The stimuli were oriented bars and their size was 0.6 degree of visual angle (dva). Two sets of stimuli were created based on the amount of orientation tilt (smaller or larger than 45°). Each set contained 8 right-tilted (quantized into 5° bins) and vertically symmetrical 8 left-tilted oriented bars. Vertical and horizontal orientations were not used. The set size of a stimulus array was 11 so there were 5 different inter-target distances. Stimuli were presented on an invisible annulus subtending 4 dva eccentricity (in radius). The approximate center-to-center inter-target distances were 2.27, 4, 6.05, 7.28, and 7.92 dva.

#### Procedure

**Figure 3:**
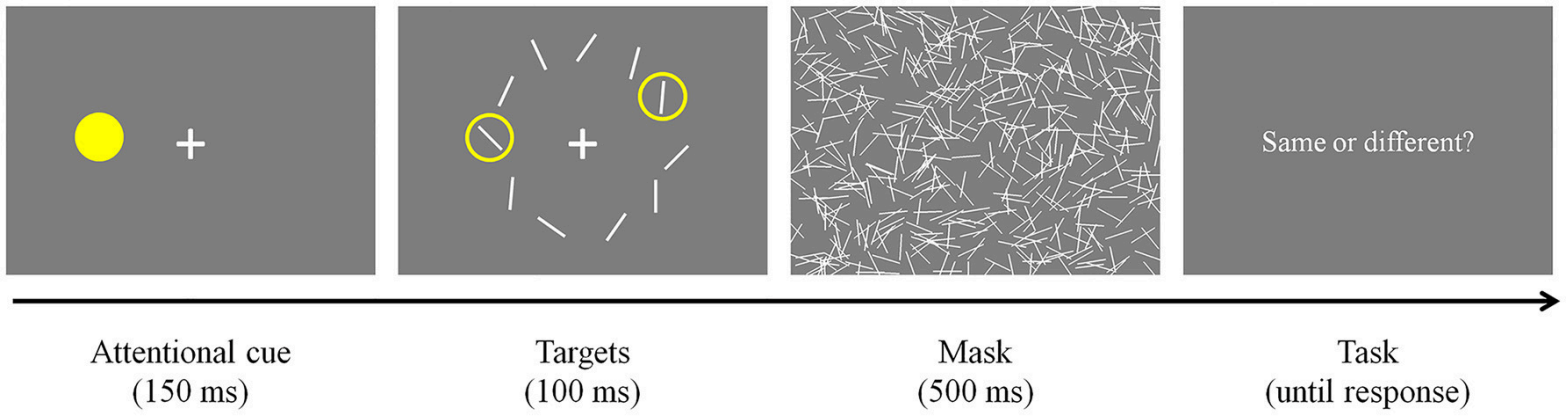
Experimental paradigm. After an attentional cue was presented for 150 ms, two target stimuli surrounded by yellow rings and distractors were presented for 100 ms. The stimulus array was masked for 500 ms to remove sensory memory. Participants responded whether the targets were the same or different by pressing buttons on a keyboard.

Participants were required to look at a fixation cross presented at the center of the display and pressed a button on a response pad when they were ready to start the task (Figure 3). Once they pressed the button and if their fixation was maintained, an attentional cue (i.e., yellow filled circle) was presented for 150 ms at a random location on an invisible annulus. Then, all stimuli were presented simultaneously and remained on the screen for 100 ms. As mentioned earlier, the spatial surround suppression appears at about 250 ms after stimulus onset (Boehler et al., 2009), and our timing choices were motivated by that result. Two of them were surrounded by yellow rings, indicating that they were the targets of the trial. The first target was always presented where the attentional cue was presented (100% valid), and the second target was presented randomly at one of the other locations. Task-irrelevant distractors were presented at the remaining locations. Target and distractor orientations were randomly selected within a stimulus set and stimulus selection was not tailored for each participant's performance. The stimulus array was followed by a 500 ms mask to remove sensory memory. Participants responded whether the two targets were the same or different by pressing buttons on the response pad. An experimental session consisted of 160 trials [10 second target locations × 2 trial types (same or different targets) × 8 repetitions, cue and first target locations were randomly selected].

## Results

**Figure 4:**
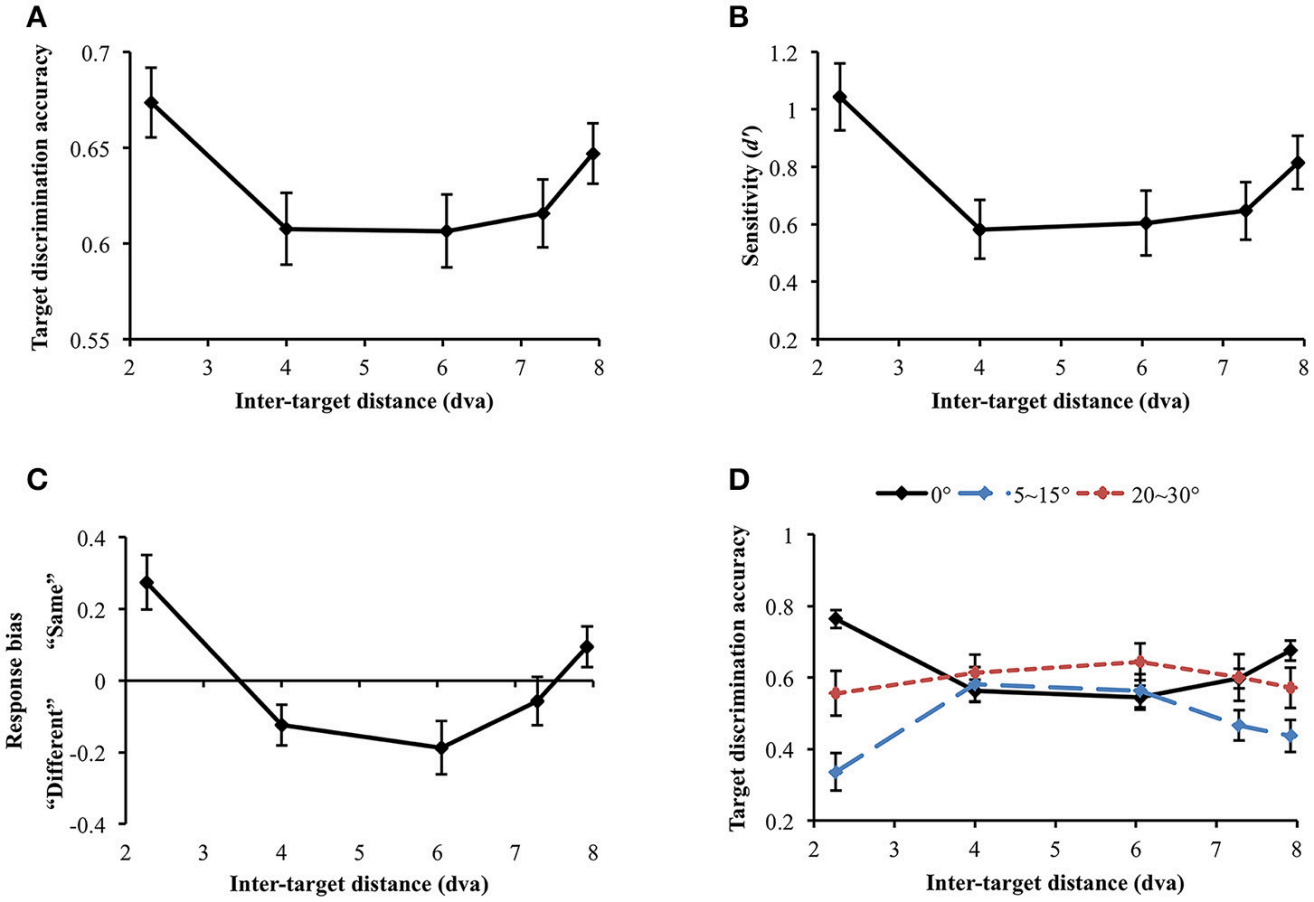
**(A)** Target discrimination accuracy (proportion correct) of Experiment 1. Accuracy was highest when the two targets were spatially closest (separated by 2.27 dva) and sharply decreased at the second closest distance (4 dva). It eventually recovered at the farthest distance (7.92 dva), demonstrating a U-shaped profile. **(B)** Participants' sensitivity for same-different discrimination (*d*′) also exhibited the comparable U-shaped profile. **(C)** Response bias changed along *d*′, demonstrating that “same” judgment, which requires thorough visual scrutiny, is associated with higher sensitivity. When attentional surround suppression plays, sensory evidence lacks so it would be more difficult to respond “same.” **(D)** Target discrimination accuracy varied depending on both orientation difference between the target stimuli and inter-target distance. When the target stimuli had the same orientation, accuracy was greatest at the closest inter-target distance, leading the U-shape profile. A typical attentional surround suppression profile (lowest accuracy at the closest inter-target distance) was observed when orientation difference between the targets was 5–15°. Surround suppression was not apparent when orientation difference became greater (20–30°). Error bars indicate SEM.

Data were not included in the analysis if target discrimination accuracy (proportion correct) was lower than 0.65 in all inter-target distance conditions (data and Matlab codes of all experiments are available at https://osf.io/xda3k). Of the 38 participants, 11 participants were excluded through this procedure [mean accuracy (SD) = 0.496 (0.037)]. Their performance was likely impaired due to short stimulus presentation duration (100 ms). For the participants able to perform the task, a repeated measures analysis of variance (ANOVA) showed that target discrimination accuracy significantly changed across inter-target distances [*F*_(4, 104)_ = 3.536, *p* = 0.010]. Accuracy sharply decreased between the first and the second closest inter-target distances [2.27 vs. 4 dva: M_diff_ = 0.067, SE = 0.026, *t*_(26)_ = 2.551, *p* = 0.017]. However, it tended to recover at the farthest inter-target distance [4 vs. 7.92 dva: M_diff_ = −0.039, SE = 0.019, *t*_(26)_ = −2.026, *p* = 0.053], showing a U-shaped profile (Figure 4A). Within individual data, 59.26% showed the U-shaped profile as average data did.

We also computed sensitivity, *d*′, to measure participants' ability to discriminate same/different targets, independently of their response bias (Green and Swets, 1966; Macmillan and Creelman, 2005). *d*′ significantly varied across inter-target distances [*F*_(4, 104)_ = 4.368, *p* = 0.003; Figure 4B]. In particular, *d*′ also exhibited the U-shaped profile as we observed in simple accuracy analysis: *d*′ diminished between the first and the second closest inter-target distances [2.27 vs. 4 dva: M_diff_ = 0.46, SE = 0.15, *t*_(26)_ = 3.01, *p* = 0.006] and then, it recovered at the farthest inter-target distance [4 vs. 7.92 dva: M_diff_ = −0.23, SE = 0.11, *t*_(26)_ = −2.142, *p* = 0.042]. This result again provides evidence for a location-based suppressive surround but in this case through a measure that is independent of response bias. Suppression of visual processing should and does decrease the sensitivity in the surround, consistent with the predictions of ST which result in reduced information in that region.

We next looked at response bias, which again showed the U-shaped profile. Response bias systematically varied along *d*′ [Greenhouse-Geisser correction (ε = 0.668): *F*_(2.67, 69.47)_ = 12.106, *p* < 0.001) and participants' tendency to respond “same” is associated with greater sensitivity (Figure 4C). In a same/different judgment, while finding differences between different stimuli is usually efficient (unless the differences are very subtle), it takes longer to confirm whether the same stimuli are identical because it involves exhaustive comparisons (Egeth, 1966; Bamber, 1969; Hawkins, 1969; Downing, 1971; Farell, 1985; Belke and Meyer, 2002). When there is limited information or visual processing of one of the stimuli, and the stimuli are identical, there will be limited evidence accumulation that it matches the template of the other stimulus. This results in a bias to categorize it as “different”, as the decision making threshold for “same” is not reached. Therefore, when attentional surround suppression is active so that information processing within the surround is constrained, it would be much more difficult to make “same” judgment. The current response bias pattern is consistent with this idea, since surround suppression reducing sensitivity produced a bias for different judgement.

Nevertheless, the discrimination accuracy and *d*′ results do not match a typical profile of the attentional suppressive surround that previous studies have shown (e.g., a monotonic profile which indicates greatest suppression when two targets are closest to each other). We wondered if an interaction between location-based and feature-based surround suppression resulted in this unusual pattern. Hence, we broke down orientation differences between the target stimuli (0°, 5~15°, and 20~30° differences) and analyzed target discrimination accuracy for each orientation difference range (Figure 4D). Note that we analyzed accuracy because we cannot calculate *d*′ after breaking down stimulus types. Target discrimination accuracy significantly varied depending on orientation difference between the target stimuli [*F*_(2, 46)_ = 12.639, *p* < 0.001], indicating the lowest accuracy when orientation difference was small (5–15°) compared to the other conditions (all *p*s' < 0.001). The main effect of inter-target distances was not significant [*F*_(4, 92)_ = 0.292, *p* = 0.882]. Interestingly, there was a significant interaction between orientation difference and inter-target distance [*F*_(8, 184)_ = 4.857, *p* < 0.001]. At the closest inter-target distance (2.27 dva), target discrimination accuracy was highest when the target orientation was the same, and it was lowest when orientation difference was 5~15° [M_diff_ = 0.428, SE = 0.063, *t*_(26)_ = 6.798, *p* < 0.001]. The accuracy was intermediate when orientation difference was 20~30°. Participants' accuracy for 0° and 5~15° orientation differences diverged again at the farthest inter-target distance [7.92 dva: M_diff_ = 0.239, SE = 0.066, *t*_(26)_ = 3.59, *p* = 0.001].

We also investigated how the profile of the attentional suppressive surround differs in each orientation difference condition. When the two targets had the same orientation, target discrimination accuracy significantly changed depending on inter-target distance [Greenhouse-Geisser correction (ε = 0.739): *F*_(2.96, 79.80)_ = 17.658, *p* < 0.001]. The accuracy was greatest at the closest inter-target distance and decreased at the second-closest inter-target distance [2.27 vs. 4 dva; M_diff_ = 20.14%, SE = 3.03%, *t*_(26)_ = 6.65, *p* < 0.001]. After then, accuracy gradually increased toward the farthest distance from the second-closest distance [*F*_(3, 78)_ = 8.04, *p* < 0.001]. This result indicated that the U-shaped profile demonstrated in the previous analysis resulted from “same target” trials. A quintessential profile of attentional surround suppression was observed when the orientation difference between the targets was small (5~15°). Target discrimination accuracy significantly changed across inter-target distances [*F*_(4, 100)_ = 5.183, *p* = 0.001], and it was lowest at the closest inter-target distance and then, improved at the second-closest inter-target distance [2.27 vs. 4 dva, M_diff_ = −24.5%, SE = 7.07%, *t*_(26)_ = 3.467, *p* = 0.002]. The same trend was maintained through the farthest distance as well [2.27 vs. 7.92 dva, M_diff_ = −10.06%, SE = 5.17%, *t*_(26)_ = 1.944, *p* = 0.063]. Lastly, target discrimination accuracy did not differ across inter-target distances when targets' orientation difference was larger [20~30°: *F*(4, 96) = 0.337, *p* = 0.852]. Consistent with our hypothesis, the current results suggest that attentional surround suppression is strongest when the stimuli are located closely to each other both in physical and feature spaces.

## Experiments 2A & 2B

As aforementioned, one theoretical prediction within ST is that the size of the attentional suppressive surround is set by the neuron's RF size that best represents the attended stimulus (Tsotsos, 2011). At its core, this links a neuron's RF size with the size of the spatial attentional suppressive surround. The original rationale for the suppressive surround was to provide a mechanism to reduce the impact of context within a RF to allow a neuron to process “signal” rather than “noise” in a more direct manner via top-down manipulation. The effect of such a mechanism has been shown previously. For example, spatial, and feature-based attention flexibly modulates neurons' RF profiles and sizes in the area MT (Womelsdorf et al., 2006, 2008; Anton-Erxleben et al., 2009; Niebergall et al., 2011), and attentional load also affects the size and the spatial tuning of population RFs in early visual cortex (V1-V3, de Haas et al., 2014). Anatomically, neurons' RFs become progressively larger as the level in the visual processing hierarchy and visual eccentricity increase (see Kay et al., 2013). A previous study examined whether the spatial extent of the surround suppression differs depending on the processing level of an attended feature (Hopf et al., 2010). The results showed that the size of the attentional suppressive surround was identical for both luminance and color targets, suggesting that the ultimate top-down selection of these features happens at the same level in visual hierarchy. However, other studies suggested that majority of neurons in early and intermediate visual areas are sensitive to both color and luminance (Gouras and Krüger, 1979; Thorell et al., 1984; Hubel and Livingstone, 1990; Lennie et al., 1990; Johnson et al., 2001; Bushnell et al., 2011), indicating that it is difficult to clearly separate the processing levels of color and luminance. Further, as mentioned earlier, it may be that the stimulus sizes and eccentricities did not make for sufficient experimental probes for this characteristic. Lastly, we hypothesized that varying the size of a stimulus would affect the size of the suppressive surround. Changes in the stimulus size influence the number of neurons that processes the stimulus within a visual processing level and then, the sum of these neurons' RFs would produce a corresponding suppressive surround. In Experiment 2, we specifically tested the effects of the size and the eccentricity of visual stimuli on the size of the suppressive surround. We used two types of stimuli (orientation and human faces in Experiment 2A and 2B, respectively) and three different stimulus size and eccentricity combinations for the target discrimination task.

### Methods and materials

#### Participants

Thirty-two (ages 18–34, 17 female) and thirty-one (ages 18–28, 17 female) York University students participated in Experiments 2A and 2B, respectively. All participants were unaware of the purpose of the experiment. They had normal or corrected-to-normal vision and normal color vision (tested with Ishihara Color Plates). Informed consent was obtained and participants recruited from Undergraduate Research Participants Pool (URPP) of York University received course credits for their study participation. The research was approved by York University's Human Participants Review Committee.

#### Apparatus and stimuli

The apparatus was the same as in Experiment 1. The stimuli used in Experiment 2A were small (1 dva) and large (3 dva) oriented bars. Except for their sizes, the stimuli were equal to those used in Experiment 1. Human faces were used in Experiment 2B (TarrLab face database: www.face-place.org; Righi et al., 2012). For human faces, only white male faces were used. All of them had short hair, the same viewpoints and facial expression, and did not wear accessories. The set size of a stimulus array was 11 so there were 5 different inter-target distances. Stimuli were presented on an invisible annulus at 7 dva eccentricity in the large and small (far) stimulus conditions, and at 4.8 dva eccentricity in the small (near) stimulus condition (in radius). The approximate center-to-center inter-stimulus distances were 3.94, 7.56, 10.58, 12.74, and 13.86 dva when stimuli were presented at 7 dva eccentricity, and 2.7, 5.2, 7.26, 8.74, and 9.5 dva when stimuli were presented at 4.8 dva eccentricity.

#### Procedure

**Figure 5:**
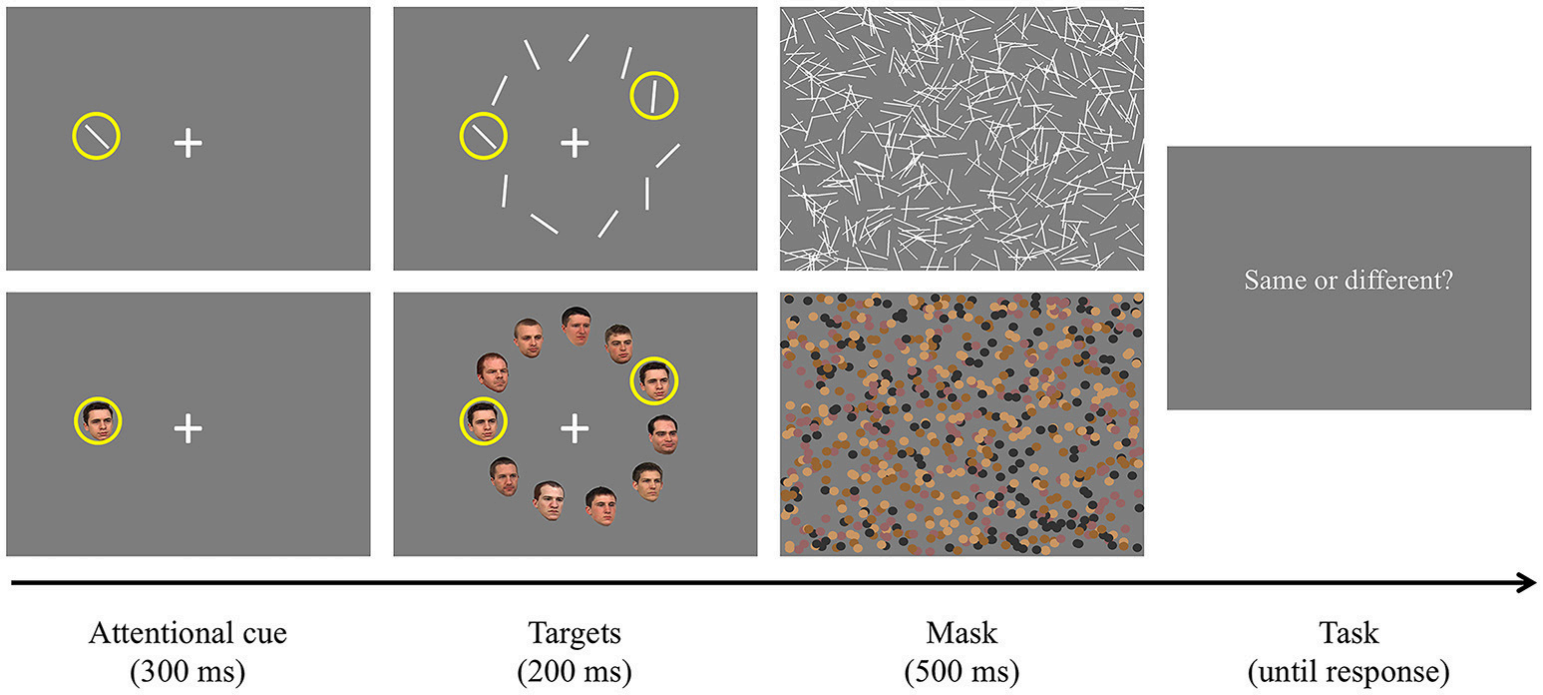
Procedure of Experiment 2. The upper panel shows an example of Experiment 2A (orientation, different targets) and the lower panel shows an example of Experiment 2B (human face, same targets). Unlike Experiment 1, one of the target stimuli surrounded by a yellow ring was presented for 300 ms as the attentional cue. Later, the other stimuli were presented for 200 ms and another target stimulus was indicated by a yellow ring. They were followed by 500 ms masking stimuli. Participants pressed a predetermined key to report whether the targets were the same or different.

The task was the same as in Experiment 1 except that one of the targets surrounded by a yellow ring was presented for 300 ms as an attentional cue (Figure 5). The ring had the same color and size as the yellow circle used in Experiment 1. We used this new attentional cue and lengthened its presentation duration to make participants focus more on the given stimulus so that they could better develop the attentional suppressive surround for it. Then, the second target and distractors were simultaneously presented for 200 ms. Since we assumed that the extremely short target presentation duration in Experiment 1 (100 ms) led to poor target discrimination accuracy, we used a longer target presentation duration in this experiment. Our pilot studies suggested that people needed much longer stimulus presentation duration specifically to perform face discrimination tasks. Half of the participants completed Experiment 2A (orientation condition) and the other half completed Experiment 2B (face condition). Within a stimulus type, there were three different display conditions [large, small (far), and small (near)]. Each display condition consisted of 160 trials [10 second target locations × 2 trial types (same or different targets) × 8 repetitions, first target locations were randomly selected].

## Results

### Experiment 2A: orientation

**Figure 6:**
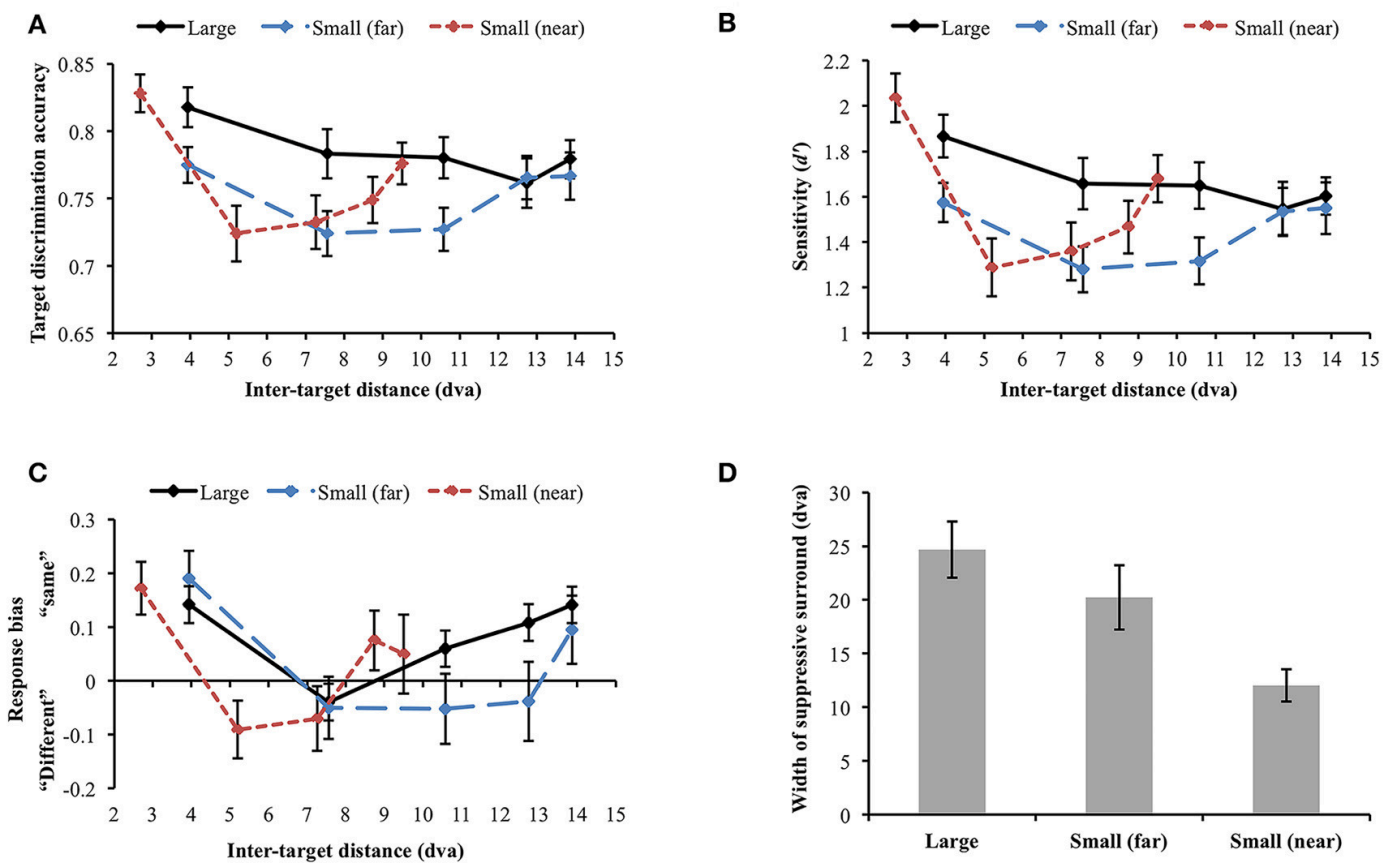
**(A)** Orientation target discrimination accuracy (proportion correct) in different stimulus size and eccentricity conditions. **(B)** The similar results were found when participants' sensitivity was analyzed. **(C)** Response bias varied along sensitivity, demonstrating an association between higher sensitivity and “same” response tendency. **(D)** Widths of the attentional suppressive surrounds. The sizes of the attentional suppressive surrounds in the large and small (far) orientation conditions did not statistically differ. Small (near) orientation condition produced a narrower suppressive surround compared to the other conditions. Error bars indicate SEM.

Data were not included in the analysis if target discrimination accuracy (proportion correct) was lower than 0.65 in all inter-target distance conditions in any of the stimulus display conditions. Through this process, data collected from 2 participants were excluded [mean accuracy (SD) = 0.454 (0.034) and 0.569 (0.146), respectively]. Since we were mainly interested in how stimulus size and eccentricity affect the attentional profile, we did not report the results after breaking down target orientation differences as in Experiment 1. However, the attentional profile in each orientation difference condition was similar across the experiments (e.g., U-shaped profile resulted from same target orientation). First, we compared target discrimination accuracy between large and small (far) orientation conditions where the stimuli were presented at the same eccentricity (Figure 6A). The main effect of stimulus size was significant [*F*_(1, 29)_ = 13.794, *p* = 0.001], indicating higher accuracy for large stimuli than small stimuli. Target discrimination accuracy was also significantly affected by inter-target distance [*F*_(4, 116)_ = 3.124, *p* = 0.018]. However, the interaction between stimulus size and inter-target distance was not significant [*F*_(4, 116)_ = 1.832, *p* = 0.127], meaning that stimulus size did not change the attentional profile. We did not compare discrimination accuracy in the small (near) condition with accuracies in the other conditions because of different inter-target distances. When each stimulus size condition was analyzed separately, target discrimination accuracy for large orientation tended to vary across different inter-target distances [*F*_(4, 116)_ = 2.101, *p* = 0.085]. Accuracy for small (far) orientation significantly varied depending on inter-target distance [*F*_(4, 116)_ = 2.835, *p* = 0.028]. It decreased at the second closest inter-target distance [3.94 vs. 7.56 dva: M_diff_ = 0.051, SE = 0.023, *t*_(29)_ = 2.269, *p* = 0.031] but it increased again at the farthest inter-target distance [7.56 vs. 13.86 dva: M_diff_ = −0.043, SE = 0.021, *t*_(29)_ = −0.2.076, *p* = 0.047], showing the U-shaped profile as found in Experiment 1. Small orientations showed a similar profile when their eccentricity had decreased [small (near)]. In the small (near) condition, target discrimination accuracy significantly varied across inter-target distance [*F*_(4, 116)_ = 8.442, *p* < 0.001]. Accuracy was significantly deteriorated at the second-closest inter-target distance [2.7 vs. 5.2 dva, M_diff_ = 0.104, SE = 0.019, *t*_(29)_ = 5.56, *p* < 0.001] but it recovered at the farthest distance [5.2 vs. 9.5 dva, M_diff_ = −0.052, SE = 0.02, *t*_(29)_ = −2.656, *p* = 0.013].

We performed a *d*′ analysis as was done in Experiment 1 (Figure 6B) and observed similar (but weaker) patterns as in the simple accuracy analysis. When large and small (far) orientation conditions were compared, large stimulus size contributed to higher d′ [*F*_(1, 29)_ = 14.493, *p* = 0.001] and *d*′ tended to vary across inter-target distance [*F*_(4, 116)_ = 2.322, *p* = 0.061]. Again, the interaction between stimulus size and inter-target distance was not significant [*F*_(4, 116)_ = 1.722, *p* = 0.15]. Changes in inter-target distance did not strongly modulate *d*′ in the large orientation condition [*F*_(4, 116)_ = 1.681, *p* = 0.159]. Nevertheless, *d*′ in the small (far) and small (near) conditions varied across different inter-target distances [small (far): *F*_(4, 116)_ = 2.401, *p* = 0.054 (marginal), small (near): *F*_(4, 116)_ = 9.788, *p* < 0.001]. In the latter two conditions, *d*′ was attenuated at the second closest inter-target distance and then improved toward the farthest distance (all *p*s < 0.05), demonstrating U-shaped profiles. Participants' tendency to respond “same” was generally associated with greater *d*′ (Figure 6C).

Next, we examined whether the size of the attentional suppressive surround is affected by stimulus eccentricity. To compare the size of the attentional suppressive surround across different display conditions, we used a curve fitting method which is more appropriate to capture the continuity of the attentional profile. ANOVA and multiple comparisons assume an independence of the suppressive effects at each inter-target distance and may not be sensitive to the continuous nature of the attentional profile. Furthermore, we cannot directly compare target discrimination accuracy through ANOVA when stimulus eccentricity differs [small (far) vs. small (near)]. As shown in Experiment 1, we observed a U-shaped attentional profile again, hence, to represent attentional profile reflected in our data, we fitted a quadratic function (i.e., U-shaped) and measured its width as an indication of the size of the attentional suppressive surround. The quadratic function explained well the average target discrimination accuracy as a function of inter-target distance [goodness-of-fit is defined by adjusted R-squared, large = 0.770; small (far) = 0.820; small (near) = 0.972]. Then, we fitted quadratic functions to individual data and measured its width (half-width at half-minimum). An outlier from the small (far) condition was excluded from the analysis (> 3 SD). Within the individual data, 56.66% of the data in the large condition, 73.33% in the small (far) condition, and 80% in the small (near) condition showed the U-shaped profile as the average data did, indicating that there was individual variability in each condition, as indicated by the variability in widths (error bars) in Figure 6D. The results (Figure 6D) showed that the attentional suppressive surrounds in the large and small (far) conditions were not statistically different [M_diff_ = 4.71 dva, SE = 3.93 dva, *t*_(28)_ = 1.2, *p* = 0.240], whereas the suppressive surround in the small (near) condition was narrower than those in the other conditions [large vs. small (near), M_diff_ = 12.65 dva, SE = 2.97 dva, *t*_(29)_ = 4.253, *p* < 0.001; small (far) vs. small (near), M_diff_ = 8.49 dva, SE = 3.41 dva, *t*_(28)_ = 2.488, *p* = 0.019].

### Experiment 2B: human faces

**Figure 7:**
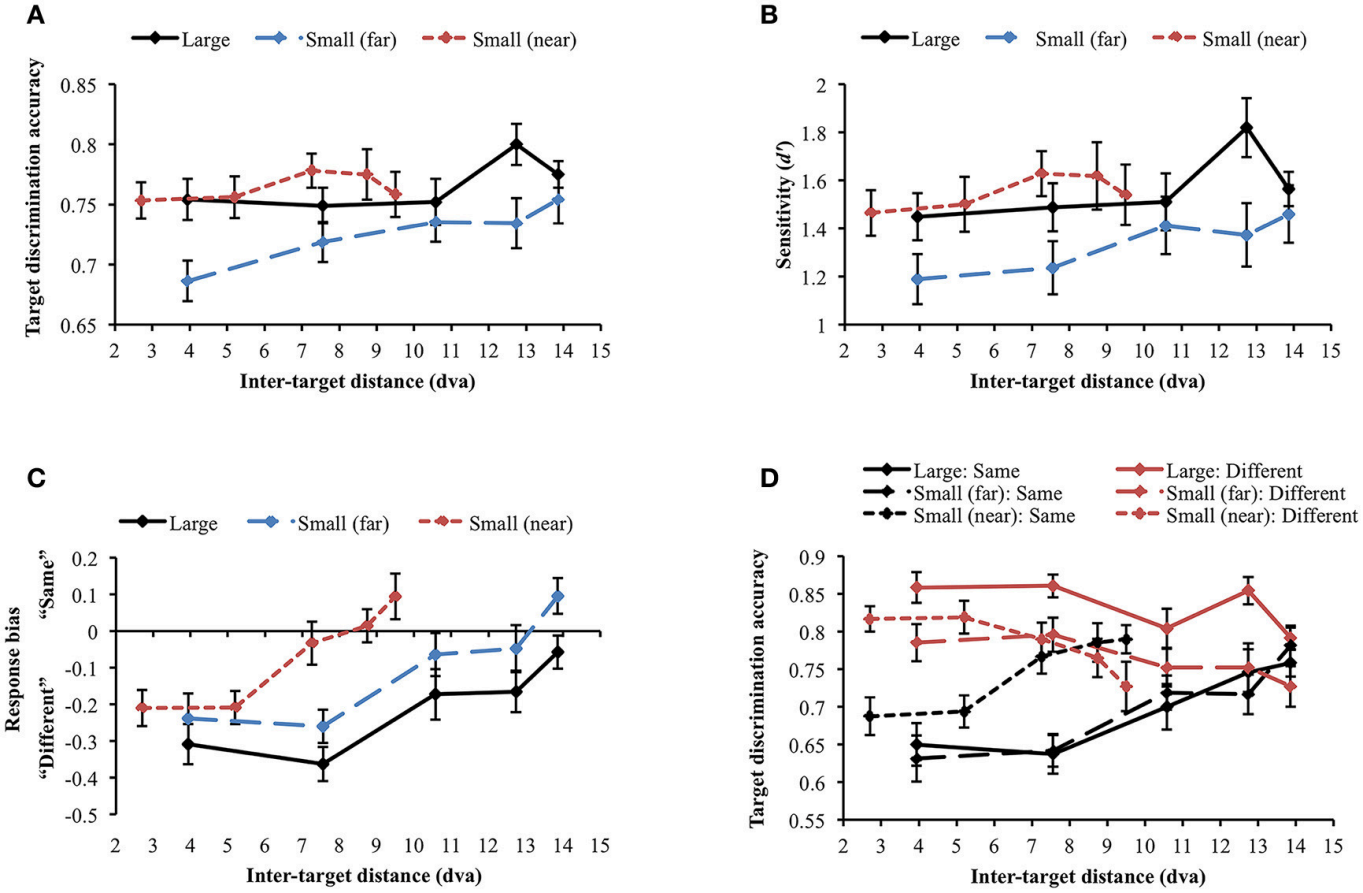
**(A)** Target discrimination accuracy and **(B)** sensitivity for face stimuli. No clear evidence for surround suppression was observed in both measures. **(C)** Participants tended to respond “same” more often as inter-target distance increased. **(D)** The identity of target faces affected target discrimination accuracy in different ways. Discrimination accuracy was greater when different faces were presented as the target stimuli (red lines), whereas it suffered when the target stimuli were the same face (black lines). Only same target faces produced attentional surround suppression. Error bars indicate SEM.

The same accuracy cut-off as in Experiment 2A was applied and one participant was excluded from the analysis through this process [mean accuracy (SD) = 0.61 (0.191)]. When the large and small (far) face conditions were compared, the main effects of stimulus size [*F*_(1, 29)_ = 6.535, *p* = 0.016] and inter-target distance [Huynh-Feldt correction (ε = 0.888): *F*_(3.55, 102.97)_ = 3.212, *p* = 0.02] were significant. However, their interaction was not significant [*F*_(4, 116)_ = 0.871, *p* = 0.484], suggesting that stimulus size did not change the profile of the attentional suppressive surround. When each display condition was separately analyzed, target discrimination accuracy significantly varied depending on inter-target distance only in the large face condition [*F*_(4, 116)_ = 2.594, *p* = 0.04] due to the accuracy peak at the second-farthest inter-target distance (8.74 dva). In contrast, accuracy remained unchanged across inter-target distances in the other conditions [small face (far): *F*_(4, 116)_ = 1.337, *p* = 0.26], [small face (near): *F*_(4, 116)_ = 0.634, *p* = 0.639], thus, they did not suggest attentional surround suppression. Figure 7A shows the results of all conditions. *d*′ analysis demonstrated the exactly same results as we found in the simple accuracy analysis (Figure 7B). In the comparison between the large and small (far) face conditions, there was an advantage of large stimulus size (*p* = 0.01) but again, the interaction between stimulus size and inter-target distance was not significant. In addition, *d*′ was modulated by inter-target distance only in the large face condition [*F*_(4, 116)_ = 2.849, *p* = 0.027] but not in the other conditions. The patterns of response bias (Figure 7C), however, indicated that participants responded “different” more often when the inter-target distance was closer, but this tendency was gradually reversed as the inter-target distance increased (in all conditions *p*s < 0.001). Based on the response bias analysis, we assumed that discrimination performance for same and different target faces would be very different across inter-target distances. As we mentioned earlier, detecting any difference between different faces is usually easier than inspecting every feature on the faces to confirm they are the same, which requires more attention. If the assumption is correct, attentional surround suppression will be produced when target faces are the same but it will be weak or even absent when target faces are different.

A repeated measures ANOVA was conducted to examine whether target identity (same vs. different) affected target discrimination accuracy (Figure 7D). First, we compared large and small (far) face conditions. The main effect of target identity on target discrimination accuracy was significant [*F*_(1, 29)_ = 26.135, *p* < 0.001], demonstrating higher accuracy when target faces were different. It supports the idea that different faces are easier to discriminate in general. Target discrimination accuracy was also affected by stimulus size [*F*_(1, 29)_ = 6.535, *p* = 0.016], indicating better performance for larger stimuli. Inter-target distance significantly changed target discrimination accuracy [Huynh-Feldt correction (ε = 0.89), *F*_(3.55, 102.97)_ = 3.21, *p* = 0.020]. The two-way interaction between target identity and stimulus size was significant [*F*_(1, 29)_ = 5.58, *p* = 0.025]. *Post-hoc* tests with Bonferroni correction showed that accuracy for large faces was higher than that for small faces only when target faces were different (M_diff_ = 0.071, SE = 0.017, *p* < 0.001) but accuracy did not differ when target faces were the same (M_diff_ = 0, SE = 0.023, *p* = 0.986). The interaction between target identity and inter-target distance [Greenhouse-Geisser correction (ε = 0.699), *F*_(2.80, 81.10)_ = 15.417, *p* < 0.001] was also significant. Target discrimination accuracy was higher in different face trials than in same face trials when inter-target distances were relatively shorter but accuracy in both same and different face trials became roughly equal at farther inter-target distances. Interaction between stimulus size and inter-target distance was not significant [*F*_(4, 116)_ = 0.871, *p* = 0.484]. Three-way interaction among target identity, stimulus size, and inter-target distance was not significant, either [*F*_(4, 116)_ = 0.100, *p* = 0.982].

Within same face trials, accuracy significantly varied across inter-target distances in all conditions [large: *F*_(4, 116)_ = 6.222, *p* < 0.001; small (far): *F*_(4, 116)_ = 8.948, *p* < 0.001; small (near): *F*_(4, 116)_ = 6.259, *p* < 0.001]. Importantly, accuracy gradually improved from the closest to the farthest inter-target distance [large: M_diff_ = 0.108, SE = 0.033, *t*_(29)_ = 3.247, *p* = 0.003; small (far): M_diff_ = 0.15, SE = 0.031, *t*_(29)_ = 4.826, *p* < 0.001; small (near): M_diff_ = 0.102, SE = 0.029, *t*_(29)_ = 3.468, *p* = 0.002]. Consistent with our hypothesis, identifying the same faces required substantial attentional processing and produced attentional surround suppression. When the target faces were different, target discrimination accuracy was significantly affected by inter-target distance for large faces [*F*_(4, 116)_ = 3.828, *p* = 0.006] and for small (near) faces [*F*_(4, 116)_ = 3.787, *p* = 0.006] but not for small (far) faces [*F*_(4, 116)_ = 1.767, *p* = 0.140]. Contrary to the accuracy patterns in same face trials, accuracy decreased between the closest and the farthest inter-target distances [large: M_diff_ = −0.067, SE = 0.021, *t*_(29)_ = −3.247, *p* = 0.003; small (far): M_diff_ = −0.058, SE = 0.029, *t*_(29)_ = 2.019, *p* = 0.053 (marginal); small (near): M_diff_ = −0.09, SE = 0.032, *t*_(29)_ = 2.842, *p* = 0.008]. It indicated that there would be no attentional surround suppression when the task is relatively easy so it does not demand much attentional processing.

Although attentional surround suppression was evident in all same face conditions, the attentional profiles quantitatively differed depending on stimulus eccentricity. In the small (near) condition, target discrimination accuracy initially improved from 0.69 to 0.77 as inter-target distance increased from 2.7 to 7.26 dva [M_diff_ = 0_._079, SE = 0.026, *t*_(29)_ = 3.072, *p* = 0.005] and then, it leveled off at farther distances [7.26 vs. 9.5 dva, M_diff_ = 0.023, SE = 0.027, *t*_(29)_ = 0.863, *p* = 0.395]. This plateau indicated the release from the surround suppression when the target stimuli were spatially well separated (Cutzu and Tsotsos, 2003). In contrast, target discrimination accuracy in the other conditions improved across the three farthest inter-target distances [10.58 vs. 13.86 dva, large: M_diff_ = 0.058, SE = 0.031, *t*_(29)_ = 1.891, *p* = 0.069 (trend level), small (far): M_diff_ = 0.063, SE = 0.029, *t*_(29)_ = 2.186, *p* = 0.037], meaning that surround suppression was still effective at this range. These results suggest that the size of the attentional suppressive surround is associated with stimulus eccentricity.

## General discussion

The present study behaviorally examined the factors that manipulate the attentional suppressive surround, which is the hallmark of the Selective Tuning model. The results demonstrated that the profile of the attentional suppressive surround is dependent on the attended spatial location, on the attended feature and on stimulus eccentricity.

### Interaction between location-based and feature-based surround suppression

Location-based and feature-based surround suppression have been studied independently so far. In Experiment 1, we found that inter-target distances and target feature similarity shape the attentional suppressive surround together, demonstrating an interaction between the two suppressive effects. Surround suppression was maximized when the target stimuli were close to each other spatially and in the feature map (i.e., when target orientations were similar). Then, it disappeared as the spatial and feature distances between the target stimuli increased. As a next step, the nature of the interaction between the two suppressive mechanisms should be addressed, such as whether they additively or multiplicatively shape the attentional tuning profile.

Unlike the typical attentional surround suppression, judging the same orientation targets was best at the closest inter-target distance, suppressed at the intermediate distances, and then recovered at farther distances. We suggest that this unexpected facilitation at the closest inter-target distance results from perceptual grouping. As proposed by Gestalt psychologists, perceptually identical and spatially adjacent stimuli tend to be grouped together and facilitated together (Koffka, 1935; Olson and Attneave, 1970; Schulz and Sanocki, 2003; Wagemans et al., 2012). However, this principle did not work on more complex features such as human faces (Experiment 2B).

One might ask if the current results could be explained by other attention models. In particular, the biased competition model (Desimone and Duncan, 1995; Reynolds et al., 1999) states that attention to one stimulus suppresses the unattended stimulus within a RF, thus it seems to predict similar location-based surround suppression. Both ST and biased competition models include attentional biases and competition among neural populations at their core. However, it should be noted that the idea of a suppressive surround due to top-down attentive localization processes first appeared in Tsotsos (1988, 1990), pre-dating biased competition and most other models of attention [for review of attention models, see Carrasco (2011), and Tsotsos (2011)]. They also differ from each other in terms of implementing suppressive mechanisms. The suppressive effects in the biased competition model result from attentional enhancement of the target stimulus, as Reynolds et al. (1999) say: “It is implemented here by increasing the efficacy of synapses projecting to the measured cell from the population activated by the attended stimulus. Increasing the strength of the signal from the attended stimulus population causes it to have a greater influence on the total mix of excitation and inhibition. Consequently, the response of the cell is driven toward the response that would be elicited if the attended stimulus were presented alone.” Unlike the biased competition model, ST does not assume attentional gain mechanisms, proposing that attentional enhancement is a consequence of an explicit top-down suppressive mechanism. This top-down suppression involves a competitive process that inhibits irrelevant signals around the attended signal which in turn, leads to surround suppression. ST specifically formulates this process, showing how the potentially suppressed signals can be determined. In addition, the attentional surround suppression is an obligatory mechanism that happens even without distractors around the target stimulus (Boehler et al., 2011), whereas suppression in the biased competition requires multiple competing stimuli. Another fundamental difference is that ST and biased competition models imply the opposite direction of attentional modulation along visual hierarchy. While the biased competition model weights attended and unattended input signals in early visual areas (V2/V4, Reynolds et al., 1999), attentional modulation targets the higher-order areas first and then moves down to the lower level in ST as supported by several studies (Lauritzen et al., 2009; e.g., Boehler et al., 2009; Buffalo et al., 2010). The biased competition model does not currently include critical components related to attentional surround suppression as it instead focuses on selection within a neuron's receptive field, while ST does predict a center-surround attentional profile (see Rothenstein and Tsotsos, 2014 for the detailed comparisons between ST and other attention models).

When it comes to feature-based surround suppression, biased competition and ST predict different outcomes. While ST predicts a center-surround attentional profile of feature-based attention (Loach et al., 2008; Tombu and Tsotsos, 2008; Störmer and Alvarez, 2014; Kiyonaga and Egner, 2016; Bartsch et al., 2017), biased competition predicts a monotonic attentional modulation depending on feature similarity between the attended and unattended features. For instance, one recent biased competition attention model (Beuth and Hamker, 2015) implements feature-based attention as a top-down amplification signal. In their simulation, however, feature-based suppression varied as a linear function of feature similarity, which contrasts with the findings of current feature-based surround suppression. In addition, Miconi and VanRullen (2016) proposed an attention model based on feedback connections and mutual inhibition within a visual area, which is related to ST. A critical difference between their model and ST is that, as the authors explicitly pointed out, this model does not assume winner-take-all processes that prune away irrelevant connections around the attended input. Thus, their model does not support spatial surround suppression, let alone feature surround suppression, which are two key tenets of ST tested in the current study. Overall, the key factor of feature surround suppression predicted by ST has been supported empirically by the current study. Other attentional models may be modified in the future to incorporate that function.

### Attentional suppressive surround is affected by stimulus eccentricity but not by stimulus size

Increments in stimulus size activate more neurons within a visual processing level, each representing a fragment of the stimulus. We hypothesized as the sum of these neurons' RFs increases, it would also enlarge the spatial extent of the attentional suppressive surround. In Experiment 2, however, stimulus size did not change the size of the attentional suppressive surround for both orientation and human faces. One possibility is that because we presented both small and large stimuli at the peripheral visual field (7 dva), the RF size at this eccentricity was already large enough to represent them as whole objects. Neurons' RF size in area V4 is around ~5 dva when visual eccentricity is 7 dva (Kay et al., 2013). In this case, both our small and large stimuli fit within the same expected RF size at this eccentricity. This result is linked to the idea that the size of the suppressive surround would be correlated with stimulus eccentricity. Our results showed that stimuli presented closer to the fovea produced a narrower suppressive surround when stimulus size was constant. RF size differences across visual eccentricity are manifested throughout visual processing hierarchy (Winawer et al., 2010; Kay et al., 2013). This means that the attended feature, independent of its processing level, would produce different suppressive surrounds in size depending on its eccentricity. One might argue that lower visibility at farther visual eccentricity could affect the current results. Nevertheless, both eccentricity conditions (near vs. far) showed roughly equal levels of target discrimination accuracy when surround suppression was maximized [mean accuracy, orientation: 72.4%, face (same): 65.94%] and when it disappeared [orientation: 77.14%, face (same): 78.54%]. Hence, visibility differences across visual eccentricity do not seem to strongly affect the results. One limitation of our stimulus design is that it confounds spacing and eccentricity of the stimuli so it might not capture the pure eccentricity effect on the attentional surround suppression. Future studies could study it by carefully matching low-level parameters in different experimental conditions.

### Attentional surround suppression reflects top-down selection refinement

ST proposes that attentional surround suppression occurs as a result of top-down selection processes that prune irrelevant connections that do not contribute to the representation of the attended stimulus. Consequently, this top-down propagation enhances spatial resolution of the attended stimulus and enables more precise localization by narrowing down the pass-zone throughout the visual processing hierarchy. To be more precise, the resolution is enhanced because interference (or entanglement) from the context of an attended stimulus is reduced. It leads to a novel hypothesis that attentional surround suppression would not be produced if a task can be performed without the need of such enhanced resolution (in location or in feature dimensions). Boehler et al. (2009) measured MEG responses during an orientation discrimination task which requires attentional focusing, and during a simple color discrimination task that can be done pre-attentively. Consistent with the hypothesis, only the orientation discrimination task elicited attentional surround suppression. In our Experiment 2B, we observed attentional surround suppression when the target faces were identical but not when they were different. As in the orientation discrimination task, one must examine features on the faces carefully to confirm they are actually the same and it requires thorough attentional focusing. On the other hand, discrimination of different faces demands relatively lesser attentional focusing. Even though we controlled some features across different faces (e.g., races, emotion, perspectives, accessories, etc.), there could be still distinctive features on the faces that made the task much easier. These results indicate that the top-down selection processes result in attentional surround suppression as ST suggests.

## Conclusions

After the Selective Tuning model proposed the existence of attentional surround suppression, many studies have reported the properties of this phenomenon using various methods. The current study provides new findings, showing that location-based and feature-based attentional surround suppression operate simultaneously to precisely demarcate the stimulus-of-interest from irrelevant distractors. It also demonstrates that the spatial extent of the suppressive surround varies by stimulus eccentricity, representing a correlation between neurons' RF size and a suppressive surround. It would be worthwhile to explore whether the present results can be replicated by varying the types of visual stimuli or even perceptual modalities. In that way, we could generalize these findings across different cognitive domains and show whether the center-surround distribution of attention is an overarching mechanism that mediates information processing in the human brain. In addition, investigating the characteristics of attentional surround suppression will have a significant impact on practical applications as well, such as UI layout development.

## Author contributions

All authors contributed to the development of the hypotheses, design of the experiments, and analytical methods. SY collected and analyzed the data, and wrote the manuscript. JT and MF provided feedback and revisions to the manuscript.

### Conflict of interest statement

The authors declare that the research was conducted in the absence of any commercial or financial relationships that could be construed as a potential conflict of interest.

## References

[B1] Adesnik H. Bruns W. Taniguchi H. Huang Z. J. Scanziani M. (2012). A neural circuit for spatial summation in visual cortex. Nature 490, 226–231. 10.1038/nature11526 23060193 PMC3621107

[B2] Andersen G. J. Kramer A. F. (1993). Limits of focused attention in three-dimensional space. Atten. Percept. Psychophys. 53, 658–667. 10.3758/BF03211742 8332432

[B3] Anton-Erxleben K. Stephan V. M. Treue S. (2009). Attention reshapes center-surround receptive field structure in macaque cortical area MT. Cereb. Cortex 19, 2466–2478. 10.1093/cercor/bhp002 19211660 PMC2742598

[B4] Bamber D. (1969). Reaction times and error rates for “same”-“different” judgments of multidimensional stimuli. Atten. Percept. Psychophys. 6, 169–174. 10.3758/BF03210087

[B5] Bartsch M. V. Loewe K. Merkel C. Heinze H. J. Schoenfeld M. A. Tsotsos J. K.. (2017). Attention to color sharpens neural population tuning via feedback processing in the human visual cortex hierarchy. J. Neurosci. 37, 10346–10357. 10.1523/JNEUROSCI.0666-17.2017 28947573 PMC6596623

[B6] Belke E. Meyer A. S. (2002). Tracking the time course of multidimensional stimulus discrimination: analyses of viewing patterns and processing times during “same”-“different” decisions. Eur. J. Cogn. Psychol. 14, 237–266. 10.1080/09541440143000050

[B7] Beuth F. Hamker F. H. (2015). A mechanistic cortical microcircuit of attention for amplification, normalization and suppression. Vis. Res. 116, 241–257. 10.1016/j.visres.2015.04.004 25883048

[B8] Boehler C. N. Tsotsos J. K. Schoenfeld M. A. Heinze H. J. Hopf J. M. (2009). The center-surround profile of the focus of attention arises from recurrent processing in visual cortex. Cereb. Cortex 19, 982–991. 10.1093/cercor/bhn139 18755778

[B9] Boehler C. N. Tsotsos J. K. Schoenfeld M. A. Heinze H. J. Hopf J. M. (2011). Neural mechanisms of surround attenuation and distractor competition in visual search. J. Neurosci. 31, 5213–5224. 10.1523/JNEUROSCI.6406-10.2011 21471356 PMC6622702

[B10] Brainard D. H. (1997). The psychophysics toolbox. Spat. Vis. 10, 433–436. 10.1163/156856897X00357 9176952

[B11] Braun J. Koch C. Davis J. L. (Eds.). (2001). Visual Attention and Cortical Circuits. Cambridge, MA: MIT Press.

[B12] Buffalo E. A. Fries P. Landman R. Liang H. Desimone R. (2010). A backward progression of attentional effects in the ventral stream. Proc. Natl. Acad. Sci. U.S.A. 107, 361–365. 10.1073/pnas.0907658106 20007766 PMC2806732

[B13] Bushnell B. N. Harding P. J. Kosai Y. Bair W. Pasupathy A. (2011). Equiluminance cells in visual cortical area V4. J. Neurosci. 31, 12398–12412. 10.1523/JNEUROSCI.1890-11.2011 21880901 PMC3171995

[B14] Bylinskii Z. DeGennaro E. M. Rajalingham R. Ruda H. Zhang J. Tsotsos J. K. (2015). Towards the quantitative evaluation of visual attention models. Vis. Res. 116, 258–268. 10.1016/j.visres.2015.04.007 25951756

[B15] Carrasco M. (2011). Visual attention: the past 25 years. Vis. Res. 51, 1484–1525. 10.1016/j.visres.2011.04.012 21549742 PMC3390154

[B16] Cheal M. L. Lyon D. R. Gottlob L. R. (1994). A framework for understanding the allocation of attention in location-precued discrimination. Q. J. Exp. Psychol. 47, 699–739. 10.1080/14640749408401134

[B17] Cutzu F. Tsotsos J. K. (2003). The selective tuning model of attention: psychophysical evidence for a suppressive annulus around an attended item. Vis. Res. 43, 205–219. 10.1016/S0042-6989(02)00491-1 12536142

[B18] de Haas B. Schwarzkopf D. S. Anderson E. J. Rees G. (2014). Perceptual load affects spatial tuning of neuronal populations in human early visual cortex. Curr. Biol. 24, R66–R67. 24456976 10.1016/j.cub.2013.11.061 PMC3928995

[B19] Denning P. J. (2007). Computing is a natural science. Commun. ACM. 50, 13–18. 10.1145/1272516.1272529

[B20] Desimone R. Duncan J. (1995). Neural mechanisms of selective attention. Annu. Rev. Neurosci. 18, 193–222. 10.1146/annurev.ne.18.030195.001205 7605061

[B21] Downing B. D. (1971). Response probabilities and “same-different” reaction times. Atten. Percept. Psychophys. 9, 213–215. 10.3758/BF03212631

[B22] Egeth H. E. (1966). Parallel versus serial processes in multidimensional stimulus discrimination. Atten. Percept. Psychophys. 1, 245–252. 10.3758/BF03207389

[B23] Eriksen C. W. Hoffman J. E. (1973). The extent of processing of noise elements during selective encoding from visual displays. Atten. Percept. Psychophys. 14, 155–160. 10.3758/BF03198630

[B24] Eriksen C. W. James J. D. (1986). Visual attention within and around the field of focal attention: a zoom lens model. Atten. Percept. Psychophys. 40, 225–240. 10.3758/BF03211502 3786090

[B25] Eriksen C. W. Yeh Y. Y. (1985). Allocation of attention in the visual field. J. Exp. Psychol. Hum. Percept. Perform. 11, 583–597. 10.1037/0096-1523.11.5.583 2932532

[B26] Farell B. (1985). “Same”–“different” judgments: a review of current controversies in perceptual comparisons. Psychol. Bull. 98, 419–456. 10.1037/0033-2909.98.3.419 4080894

[B27] Gouras P. Krüger J. (1979). Responses of cells in foveal visual cortex of the monkey to pure color contrast. J. Neurophysiol. 42, 850–860. 10.1152/jn.1979.42.3.850 107286

[B28] Green D. Swets J. (1966). Signal Detection Theory and Psychophysics. New York, NY: Wiley.

[B29] Haider B. Krause M. R. Duque A. Yu Y. Touryan J. Mazer J. A.. (2010). Synaptic and network mechanisms of sparse and reliable visual cortical activity during nonclassical receptive field stimulation. Neuron 65, 107–121. 10.1016/j.neuron.2009.12.005 20152117 PMC3110675

[B30] Hawkins H. L. (1969). Parallel processing in complex visual discrimination. Atten. Percept. Psychophys. 5, 56–64. 10.3758/BF03210482

[B31] Hopf J. M. Boehler C. N. Luck S. J. Tsotsos J. K. Heinze H. J. Schoenfeld M. A. (2006). Direct neurophysiological evidence for spatial suppression surrounding the focus of attention in vision. Proc. Natl. Acad. Sci. U.S.A. 103, 1053–1058. 10.1073/pnas.0507746103 16410356 PMC1347985

[B32] Hopf J. M. Boehler C. N. Schoenfeld M. A. Heinze H. J. Tsotsos J. K. (2010). The spatial profile of the focus of attention in visual search: insights from MEG recordings. Vision Res. 50, 1312–1320. 10.1016/j.visres.2010.01.015 20117126

[B33] Hubel D. H. Livingstone M. S. (1990). Color and contrast sensitivity in the lateral geniculate body and primary visual cortex of the macaque monkey. J. Neurosci. 10, 2223–2237. 10.1523/JNEUROSCI.10-07-02223.1990 2198331 PMC6570379

[B34] Hubel D. H. Wiesel T. N. (1968). Receptive fields and functional architecture of monkey striate cortex. J. Physiol. 195, 215–243. 10.1113/jphysiol.1968.sp008455 4966457 PMC1557912

[B35] Hubel D. H. Wiesel T. N. (1974). Uniformity of monkey striate cortex: a parallel relationship between field size, scatter, and magnification factor. J. Comp. Neurol. 158, 295–305. 10.1002/cne.901580305 4436457

[B36] Johnson E. N. Hawken M. J. Shapley R. (2001). The spatial transformation of color in the primary visual cortex of the macaque monkey. Nat. Neurosci. 4, 409–416. 10.1038/86061 11276232

[B37] Kay K. N. Winawer J. Mezer A. Wandell B. A. (2013). Compressive spatial summation in human visual cortex. J. Neurophysiol. 110, 481–494. 10.1152/jn.00105.2013 23615546 PMC3727075

[B38] Kiyonaga A. Egner T. (2016). Center-surround inhibition in working memory. Curr. Biol. 26, 64–68. 10.1016/j.cub.2015.11.013 26711496 PMC4713284

[B39] Koffka K. (1935). Principles of Gestalt Psychology. New York, NY: Harcourt, Brace & World.

[B40] LaBerge D. (1983). Spatial extent of attention to letters and words. J. Exp. Psychol. Hum. Percept. Perform. 9, 371–379. 10.1037/0096-1523.9.3.371 6223977

[B41] LaBerge D. Brown V. (1986). Variations in size of the visual field in which targets are presented: an attentional range effect. Atten. Percept. Psychophys. 40, 188–200. 10.3758/BF03203016 3774503

[B42] Lauritzen T. Z. D'Esposito M. Heeger D. J. Silver M. A. (2009). Top–down flow of visual spatial attention signals from parietal to occipital cortex. J. Vis. 9:18. 10.1167/9.13.18 20055551 PMC2857595

[B43] Lennie P. Krauskopf J. Sclar G. (1990). Chromatic mechanisms in striate cortex of macaque. J. Neurosci. 10, 649–669. 10.1523/JNEUROSCI.10-02-00649.1990 2303866 PMC6570166

[B44] Loach D. Frischen A. Bruce N. Tsotsos J. K. (2008). An attentional mechanism for selecting appropriate actions afforded by graspable objects. Psychol. Sci. 19, 1253–1257. 10.1111/j.1467-9280.2008.02234.x 19121133

[B45] Macmillan N. A. Creelman C. D. (2005). Detection Theory: A User's Guide, 2nd Edn. Mahwah, NJ: Erlbaum.

[B46] Miconi T. VanRullen R. (2016). A feedback model of attention explains the diverse effects of attention on neural firing rates and receptive field structure. PLoS Comput. Biol. 12:e1004770. 10.1371/journal.pcbi.1004770 26890584 PMC4758641

[B47] Müller N. G. Kleinschmidt A. (2004). The attentional “spotlight's” penumbra: center-surround modulation in striate cortex. Neuroreport 15, 977–980. 10.1097/00001756-200404290-00009 15076718

[B48] Müller N. G. Mollenhauer M. Rösler A. Kleinschmidt A. (2005). The attentional field has a Mexican hat distribution. Vision Res. 45, 1129–1137. 10.1016/j.visres.2004.11.003 15707921

[B49] Niebergall R. Khayat P. S. Treue S. Martinez-Trujillo J. C. (2011). Expansion of MT neurons excitatory receptive fields during covert attentive tracking. J. Neurosci. 31, 15499–15510. 10.1523/JNEUROSCI.2822-11.2011 22031896 PMC6703514

[B50] Olson R. K. Attneave F. (1970). What variables produce similarity grouping. Am. J. Psychol. 83, 1–21. 10.2307/1420852

[B51] Ozeki H. Finn I. M. Schaffer E. S. Miller K. D. Ferster D. (2009). Inhibitory stabilization of the cortical network underlies visual surround suppression. Neuron 62, 578–592. 10.1016/j.neuron.2009.03.028 19477158 PMC2691725

[B52] Pelli D. G. (1997). The videotoolbox software for visual psychophysics: transforming numbers into movies. Spat. Vis. 10, 437–442. 10.1163/156856897X00366 9176953

[B53] Posner M. I. Snyder C. R. Davidson B. J. (1980). Attention and the detection of signals. J. Exp. Psychol. Gen. 109, 160–174. 10.1037/0096-3445.109.2.160 7381367

[B54] Reynolds J. H. Chelazzi L. Desimone R. (1999). Competitive mechanisms subserve attention in macaque areas V2 and V4. J. Neurosci. 19, 1736–1753. 10.1523/JNEUROSCI.19-05-01736.1999 10024360 PMC6782185

[B55] Righi G. Peissig J. J. Tarr M. J. (2012). Recognizing disguised faces. Vis. Cogn. 20, 143–169. 10.1080/13506285.2012.654624

[B56] Rothenstein A. L. Tsotsos J. K. (2014). Attentional modulation and selection–an integrated approach. PLoS ONE 9:e99681. 10.1371/journal.pone.0099681 24963827 PMC4070899

[B57] Schulz M. F. Sanocki T. (2003). Time course of perceptual grouping by color. Psychol. Sci. 14, 26–30. 10.1111/1467-9280.01414 12564750

[B58] Self M. W. Lorteije J. A. Vangeneugden J. van Beest E. H. Grigore M. E. Levelt C. N.. (2014). Orientation-tuned surround suppression in mouse visual cortex. J. Neurosci. 34, 9290–9304. 10.1523/JNEUROSCI.5051-13.2014 25009262 PMC6608354

[B59] Smith A. T. Singh K. D. Williams A. L. Greenlee M. W. (2001). Estimating receptive field size from fMRI data in human striate and extrastriate visual cortex. Cereb. Cortex 11, 1182–1190. 10.1093/cercor/11.12.1182 11709489

[B60] Störmer V. S. Alvarez G. A. (2014). Feature-based attention elicits surround suppression in feature space. Curr. Biol. 24, 1985–1988. 10.1016/j.cub.2014.07.030 25155510

[B61] Thorell L. G. De Valois R. L. Albrecht D. G. (1984). Spatial mapping of monkey V1 cells with pure color and luminance stimuli. Vision Res. 24, 751–769. 10.1016/0042-6989(84)90216-5 6464367

[B62] Tombu M. Tsotsos J. K. (2008). Attending to orientation results in an inhibitory surround in orientation space. Atten. Percept. Psychophys. 70, 30–35. 10.3758/PP.70.1.30 18306958

[B63] Tsotsos J. K. (1988). A “complexity level” analysis of immediate vision. Int. J. Comput. Vis. 1, 303–320. 10.1007/BF00133569

[B64] Tsotsos J. K. (1990). Analyzing vision at the complexity level. Behav. Brain Sci. 13, 423–445. 10.1017/S0140525X00079577

[B65] Tsotsos J. K. (2011). A Computational Perspective on Visual Attention. Cambridge, MA: MIT Press.

[B66] Wagemans J. Elder J. H. Kubovy M. Palmer S. E. Peterson M. A. Singh M.. (2012). A century of Gestalt psychology in visual perception: i. Perceptual grouping and figure–ground organization. Psychol. Bull. 138, 1172–1217. 10.1037/a0029333 22845751 PMC3482144

[B67] Wilson J. R. Sherman S. M. (1976). Receptive-field characteristics of neurons in cat striate cortex: changes with visual field eccentricity. J. Neurophysiol. 39, 512–533. 10.1152/jn.1976.39.3.512 948006

[B68] Winawer J. Horiguchi H. Sayres R. A. Amano K. Wandell B. A. (2010). Mapping hV4 and ventral occipital cortex: the venous eclipse. J. Vis. 10:1. 10.1167/10.5.1 20616143 PMC3033222

[B69] Womelsdorf T. Anton-Erxleben K. Pieper F. Treue S. (2006). Dynamic shifts of visual receptive fields in cortical area MT by spatial attention. Nat. Neurosci. 9, 1156–1160. 10.1038/nn1748 16906153

[B70] Womelsdorf T. Anton-Erxleben K. Treue S. (2008). Receptive field shift and shrinkage in macaque middle temporal area through attentional gain modulation. J. Neurosci. 28, 8934–8944. 10.1523/JNEUROSCI.4030-07.2008 18768687 PMC6670861

